# Erratum

**DOI:** 10.1002/cfg.390

**Published:** 2004-03


					Comparative and Functional Genomics
Comp Funct Genom 2004; 5: 205.
Published online in Wiley InterScience (www.interscience.wiley.com). DOI: 10.1002/cfg.390
Erratum
The article by Stefancsik, R., Randall, J.D., Mao, C., and Sarkar, S., Structure and sequence of the
human fast skeletal troponin T (TNNT3) gene: insight into the evolution of the gene and the origin of
the developmentally regulated isoforms, Comp. Funct. Genom. 2003: 4: 609-625 contains an error in
Table 3 on page 620. The correct version of Table 3 is shown below.
Table 3. Intron phasing of invertebrate TnT genes
Intron No./phase 1 2 3 4 5 6 7 8 9 10 11 12 13 14 15
Drosophila TnT 2 X X X
1 X
0 X X X1 X X
C. elegans TnT-1 2 X X
1
0 X X X1
C. elegans TnT-2 2 X X
1 X
0 X X1 X
C. elegans TnT-3 2 X X X X X
1 X X
0 X X X X X1 X X X
C. elegans TnT-4 2 X X
1
0 X1 X X
For details, see also text. X1, intron located in the region coding for the HR domain. Sources of DNA sequences: Drosophila TnT, Benoist et
al. (1998), Fyrberg et al. (1990); C. elegans TnT-1, Myers et al. (1996); C. elegans TnT-2, GenBank Accession No. 746561; C. elegans TnT-3,
GenBank Accession No. 861386; C. elegans TnT-4, GenBank Accession No. 2736369.
Copyright  2004 John Wiley & Sons, Ltd.

				
